# Inter-vendor and inter-observer reliability of diffusion tensor imaging in the musculoskeletal system: a multiscanner MR study

**DOI:** 10.1186/s13244-023-01374-0

**Published:** 2023-02-09

**Authors:** Vito Chianca, Domenico Albano, Stefania Rizzo, Mario Maas, Luca Maria Sconfienza, Filippo Del Grande

**Affiliations:** 1 Clinica di Radiologia EOC IIMSI, Lugano, Switzerland; 2 Ospedale Evangelico Betania, Via Argine 604, 80147 Naples, Italy; 3 IRCCS Istituto Ortopedico Galeazzi, Milan, Italy; 4 Department of Radiology and Nuclear Medicine, Amsterdam University Medical Centres, University of Amsterdam, Amsterdam, The Netherlands; 5 Amsterdam Movement Sciences Research Institute, Amsterdam, The Netherlands; 6 Department of Biomedical Sciences for Health, University of Milano, Milan, Italy

**Keywords:** Muscle, Diffusion tensor imaging, Magnetic resonance, Reliability, Reproducibility

## Abstract

**Background:**

To evaluate the inter-observer and inter-vendor reliability of diffusion tensor imaging parameters in the musculoskeletal system.

**Methods:**

This prospective study included six healthy volunteers three men (mean age: 42; range: 31–52 years) and three women (mean age: 36; range: 30–44 years).

Each subject was scanned using different 3 Tesla magnetic resonance scanners from three different vendors at three different sites bilaterally. First, the intra-class correlation coefficient was used to determine between-observers agreement for overall measurements and clinical sites. Next, between-group comparisons were made through the nonparametric Friedman’s test. Finally, the Bland–Altman method was used to determine agreement among the three scanner measurements, comparing them two by two.

**Results:**

A total of 792 measurement were calculated. ICC reported high levels of agreement between the two observers. ICC related to MD, FA, and RD measurements ranged from 0.88 (95% CI 0.85–0.90) to 0.95 (95% CI 0.94–0.96), from 0.85 (95% CI 0.81–0.88) to 0.95 (95% CI 0.93–0.96), and from 0.89 (0.85–0.90) to 0.92 (0.90–0.94).

No statistically significant inter-vendor differences were observed. The Bland–Altmann method confirmed a high correlation between parameter values.

**Conclusion:**

An excellent inter-observer and inter-vendor reliability was found in our study.

**Supplementary Information:**

The online version contains supplementary material available at 10.1186/s13244-023-01374-0.

## Key points

1. Diffusion tensor imaging shows excellent inter-observer reliability.
2. Diffusion tensor imaging shows excellent inter-vendor reliability.
3. Quantitative results in musculoskeletal application of diffusion tensor imaging are reproducible in all the three magnetic resonance scanners.
4. DTI, as a reproducible magnetic resonance sequence, can be used for a quantitative evaluation of muscle microstructures during daily practice.

**Supplementary Information:**

The online version contains supplementary material available at 10.1186/s13244-023-01374-0.

## Introduction

Since its first clinical application in the musculoskeletal (MSK) system, muscle diffusion tensor imaging (m-DTI) has become a valuable sequence for the evaluation of architectural changes of fibre microenvironments [1–4]. M-DTI provides parameters such as fractional anisotropy (FA), radial diffusivity (RD), and mean diffusivity (MD) that allow extracting quantitative information on integrity of muscle fibres [5, 6]. Inflammatory pathologies, traumatic injuries, neuromuscular disorders, or atrophic conditions are the principal areas of application of m-DTI in conjunction with conventional sequences in the assessments of early structural changes [7–11].

Furthermore, post-processing of DTI is able to generate fibre tractography and to assess 3D muscular structure from the origin to distal insertion by calculating architectural parameters such as fibre length, number and volume, and pennation angle [12].

Despite its potential to assess structural changes of the muscles, the application of m-DTI in clinical practice is still controversial because of several factors influencing DTI signals, such as field strength, gradient strength, b-values, and post-processing algorithms [13]. Several studies reported an acceptable agreement of DTI measurements on the brain, whereas m-DTI studies, which were mainly conducted on lower limb muscle group, reported relatively high variations with FA values ranging from 0.28 to 0.6 [6, 14]. Moreover, some studies on brain DTI reported conflicting results regarding inter-site, intra-site, and inter-vendor reliability [15–17]. To the best of our knowledge, no studies assess m-DTI inter-vendor agreement. The aims of our study were to assess the inter-reader reliability and the inter-vendor reliability on 3 T magnetic resonance (MR) for m-DTI measurements.

## Materials and methods

### Study subjects

The local ethics committee approved our prospective study, and all participants signed an informed consent before starting the examination. The study was conducted in compliance with the Declaration of Helsinki.

We enrolled six healthy volunteers: Three were males (mean age: 42; range: 31–52 years) and three women (mean age: 36; range: 30–44 years).

Inclusion criteria were: 18 year or older, no neuromuscular diseases in their personal and/or family history, no present or past muscle strains in the muscular group under evaluation, and no participation in any sports activity three weeks before the examination.

Exclusion criteria for enrolment were: usual contraindications to MR imaging, positive pregnancy test, and objects in the body that could obscure the target muscle groups through artefacts. After having optimized the sequences in collaboration with the specialists of the different vendors, each volunteer was scanned once on three different anatomical sites bilaterally (middle third of the arm, middle third of the leg, and middle third of the thigh). All scans were acquired on the same day within 6 h to reduce any possible bias and were checked for image quality and artefacts.

### MR examination

MR examinations were performed using three 3 T (T) MR of our institution: Signa Pioneer (GE Healthcare, Milwaukee, WI, USA), Achieva (Philips Healthcare, Best, Netherlands), and Skyra (Siemens Healthineers, Erlangen, Germany). The total MR examination time for Signa Pioneer, Achieva, and Skyra was 18.05, 17.56, and 17.52 min, respectively. MR protocol acquisition parameters including RF-coils are summarized in Tables 1 and 2.

**Table 1:** DTI acquisition parameters

	GE	Philips	Siemens
DTI parameters (Thigh)			
Coil	16 channels	32 channels	18 channels
Directions	12	15	12
b-value (s/mm^2^)	400	400	400
TR (ms)	7350	7500	7500
TE (ms)	Minimum	72	83.0
Matrix	128 × 128	128 × 128	128 × 128
Parallel factor	2 (asset)	2 (sense)	2 (grappa)
Voxel size (mm)	3.1 × 3.1 × 4.0	3.03 × 2.97 × 4.0	3.1 × 3.1 × 4.0
Acquisition time (minutes:seconds)	3.18	4.07	3.32
DTI parameters (leg)			
Coil	16 channels	32 channels	18 channels
Directions	12	12	12
b-value (s/mm^2^)	400	400	400
TR (ms)	7950	7500	7500
TE (ms)	Minimum	66	83
Matrix	128 × 128	128 × 128	128 × 128
Parallel factor	2 (asset)	2 (sense)	2 (grappa)
Voxel size (mm)	2.3 × 2.3 × 4.0	2.3 × 2.3 × 4.0	2.3 × 2.3 × 4.0
Acquisition time (minutes:seconds)	3.35	4.07	3.32
DTI parameters (arm)			
Coil	16 channels	8 channels	18 channels
Directions	12	12	12
b-value (s/mm^2^)	400.0	400	400
TR (ms)	8600	7837	7900
TE (ms)	Minimum	79	83
Matrix	122 × 122	122 × 122	122 × 122
Parallel factor	2 (asset)	2 (sense)	2 (grappa)
Voxel size (mm)	1.6 × 1.6 × 4.0	1.6 × 1.6 × 4.0	1.6 × 1.6 × 4.0
Acquisition time (minutes: seconds)	3.52	4.18	3.43

**Table 2:** T1 TSE acquisition parameters

	GE	Philips	Siemens
Thigh			
TR (ms)	604	519	600
TE (ms)	Minimum	13	11
Matrix	448	448	448
Parallel factor	2 (asset)	(2 sense)	2 (grappa)
Voxel size (mm)	0.9 × 0.9 × 4.0	0.9 × 0.9 × 4	0.9 × 0.9 × 4.0
Acquisition time (minutes: seconds)	2.01	1.36	1.52
Leg			
TR (ms)	618	519	600
TE (ms)	Minimum	13	11
Matrix	384 × 384	384 × 384	384 × 384
Parallel factor	2 (asset)	2 (sense)	2 (grappa)
Voxel size (mm)	0.8 × 0.8 × 4.0	0.8 × 0.8 × 4.0	0.8 × 0.8 × 4.0
Acquisition time (minutes: seconds)	1.49	1.33	1.38
Arm			
TR (ms)	635.0	519	605.0
TE (ms)	Minimum	13	11.0
Matrix	256 × 256	256 × 256	256 × 256
Parallel factor	2 (asset)	2 (sense)	2 (grappa)
Voxel size (mm)	0.7 × 0.7 × 4.0	0.7 × 0.7 × 4.0	0.7 × 0.7 × 4.0
Acquisition time (minutes: seconds)	1.40	1.25	1.29

### Image analysis

**Fig. 1:**
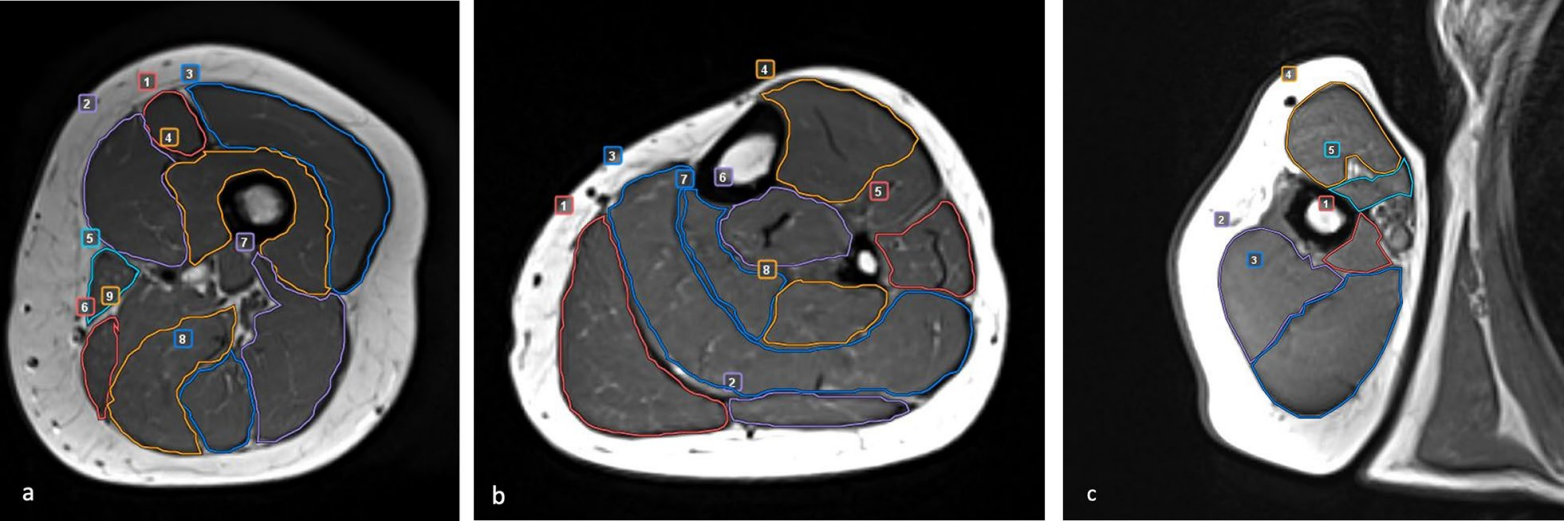
Axial T1w images showing ROIs of the different anatomical compartments. **a** 1 rectus femoris, 2 vastus medialis, 3 vastus lateralis, 4 vastus intermedius, 5 sartorius, 6 gracilis, 7 biceps femoris, 8 semitendinosus, 9 semimembranosus. **b** 1 Medial gastrocnemius, 2 lateral gastrocnemius, 3 soleus, 4 anterior tibialis, 5 peroneal muscles, 6 posterior tibialis, 7 flexor digitorum longus, 8 flexor hallucis longus. **c** 1 Medial head of triceps brachii, 2 lateral head of triceps brachii, 3 long head of triceps brachii, 4 biceps brachii, and 5 coraco brachialis

**Fig. 2:**
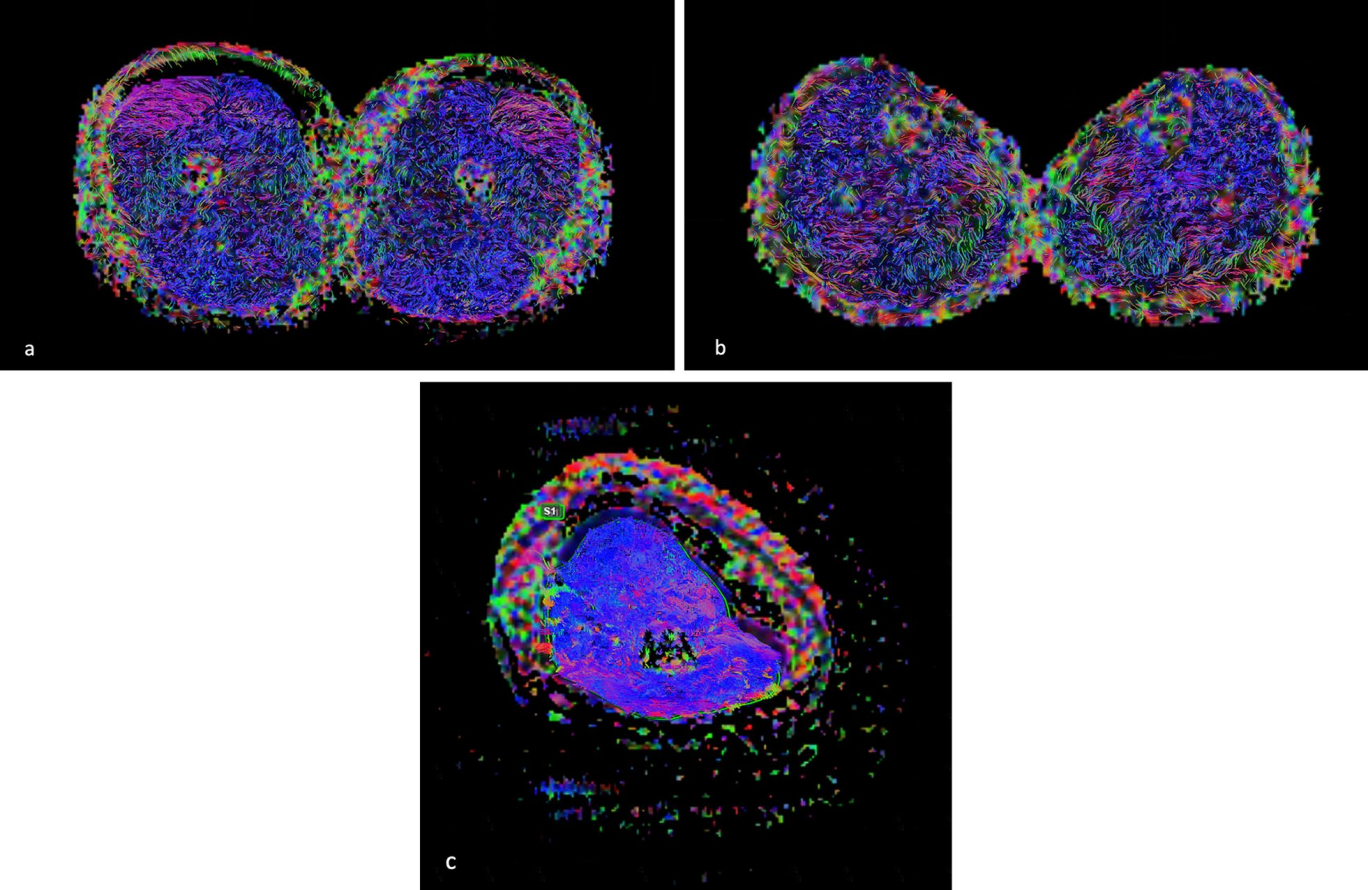
Axial color FA map with overlaid tractography of the thighs (**a**), legs (**b**), and arm (**c**)

Following data acquisition and after removing all patient identifying information, a radiologist with eight years of experience in MSK MR interpretation assessed image quality [18]. Then, m-DTI parameters on different muscle compartments were independently assessed by two radiologists (8 and 10 years of experience in the MSK field) using a commercially available software (Olea sphere 3.0). The muscle regions of interest (ROIs) were selected as described in Fig. 1. Post-processing was performed on the DTI images. Motion-related misalignments and adjacent image noise were corrected with automated image registration. Both readers manually drew the ROIs on the same slices at the middle third of the thigh, leg, and arm on axial T1w sequences as shown in Fig. 1. FA, RD, and MD values of the different muscle areas were calculated. Fibre tractography of the thigh, leg, and arm is shown in Fig. 2.

### Statistical analysis

Results are reported as medians and interquartile ranges (IQR). Data distributions were checked for normality using the Shapiro–Wilk test, which showed that all data were non-normally distributed (*p* < 0.05). Next, the intra-class correlation coefficient (ICC) was used to determine between-observers agreement for overall measurements and clinical sites, keeping in this last case, the distinction between left and right side, so to use the contralateral side as a double check of agreement. Next, between-group comparisons were made through the nonparametric Friedman’s test. We applied Friedman’s test for each observer’s measurements to prevent biased findings. Finally, the Bland–Altman method was used to determine agreement among the three scanner measurements, comparing them two by two. A *p* value less than 0.01 was considered to be statistically significant. Statistical analyses were executed by MedCalc Statistical Software version 19.2.6 (MedCalc Software bv, Ostend, Belgium; https://www.medcalc.org; 2020).

## Results

### Agreement between observers

The ICC reported high levels of agreement between the two observers as summarized in Table 3.

**Table 3:** Intra-class correlation

	GE	Philips	Siemens
MD	0.95 (0.94–0.96)	0.92 (0.90–0.94)	0.88 (0.85–0.90)
FA	0.91 (0.88–0.93)	0.95 (0.93–0.96)	0.85 (0.81–0.88)
RD	0.89 (0.86–0.91)	0.92 (0.90–0.94)	0.89 (0.85–0.90)

Good to excellent ICC values (higher than 0.69) were assessed between the two observers according to anatomical sites, except for FA measurement on Siemens MR (0.62, 95% CI 0.43–0.76). Detailed results are reported in Table 4.

**Table 4:** ICC for clinical sites

	GE	Philips	Siemens
MD			
Right arm	0.97 (0.95–0.99)	0.89 (0.78–0.95)	0.76 (0.56–0.88)
Left arm	0.96 (0.91–0.98)	0.96 (0.93–0.98)	0.69 (0.44–0.84)
Right thigh	0.92 (0.87–0.95)	0.94 (0.90–0.96)	0.94 (0.89–0.96)
Left thigh	0.94 (0.89–0.96)	0.86 (0.77–0.91)	0.97 (0.94–0.98)
Right leg	0.94 (0.91–0.97)	0.96 (0.93–0.98)	0.91 (0.84–0.95)
Left leg	0.96 (0.94–0.98)	0.88 (0.79–0.93)	0.92 (0.86–0.95)
FA			
Right arm	0.96 (0.92–0.98)	0.86 (0.73–0.93)	0.88 (0.76–0.94)
Left arm	0.71 (0.49–0.85)	0.92 (0.83–0.96)	0.94 (0.87–0.97)
Right thigh	0.93 (0.88–0.96)	0.96 (0.93–0.98)	0.62 (0.43–0.76)
Left thigh	0.97 (0.94–0.98)	0.94 (0.90–0.96)	0.96 (0.94–0.98)
Right leg	0.86 (0.76–0.92)	0.93 (0.89–0.96)	0.93 (0.88–0.96)
Left leg	0.93 (0.88–0.96)	0.95 (0.92–0.97)	0.87 (0.77–0.92)
RD			
Right arm	0.98 (0.95–0.99)	0.89 (0.78–0.94)	0.84 (0.69–0.92)
Left arm	0.96 (0.93–0.98)	0.96 (0.92–0.98)	0.99 (0.98–0.99)
Right thigh	0.92 (0.86–0.95)	0.93 (0.89–0.96)	0.92 (0.87–0.95)
Left thigh	0.93 (0.89–0.96)	0.87 (0.79–0.92)	0.97 (094–0.98)
Right leg	0.95 (0.90–0.97)	0.94 (0.90–0.97)	0.90 (0.83–0.94)
Left leg	0.96 (0.93–0.98)	0.87 (0.78–0.93)	0.89 (0.81–0.94)

### Inter-vendor reliability

No statistically significant inter-vendor differences were observed for both readers and for all the parameters. (Table 5). MD measurements for reader two were close to significance (*p* = 0.0573).

**Table 5:** Inter-vendor differences in MD, FA, and RD

	GE	Philips	Siemens	*p* value*
	Median (IQR)	Median (IQR)	Median (IQR)	
1° Observer				
MD	1.563 (0.295)	1.533 (0.253)	1.571 (0.346)	0.1152
FA	0.303 (0.059)	0.299 (0.071)	0.300 (0.072)	0.3772
RD	1.319 (0.257)	1.301 (0.252)	1.340 (0.318)	0.1643
CV of MD	0.17	0.14	0.23	–
CV of FA	0.15	0.16	0.17	–
CV of RD	0.18	0.15	0.23	–
2° Observer				
MD	1.559 (0.299)	1.541 (0.264)	1.592 (0.356)	0.0573
FA	0.301 (0.059)	0.300 (0.069)	0.300 (0.067)	0.7046
RD	1.320 (0.283)	1.312 (0.258)	1.343 (0.344)	0.3258
CV of MD	0.18	0.14	0.23	–
CV of FA	0.15	0.16	0.19	–
CV of RD	0.18	0.14	0.25	–

*p value was determined through Friedman’s Test

*MD* mean diffusivity, *RA* radial anisotropy, *FA* fractional anisotropy, *RD* radial diffusivity, *CV* coefficient of variation

**Fig. 3:**
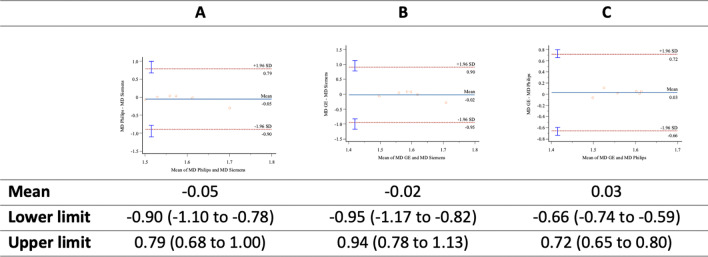
MD-**A**: Philips versus Siemens; **B**: GE versus Siemens; **C**: GE versus Philips

**Fig. 4:**
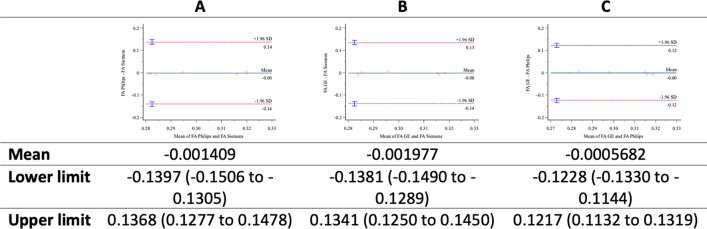
FA-**A**: Philips versus Siemens; **B**: GE versus Siemens; **C**: GE versus Philips

**Fig. 5:**
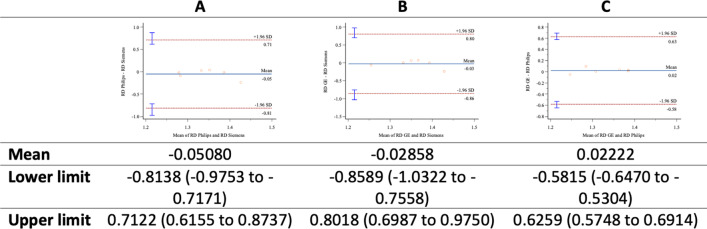
RD-**A**: Philips versus Siemens; **B** GE versus Siemens; **C** GE versus Philips

Bland–Altman plots comparing MD (Fig. 3), FA (Fig. 4), and RD (Fig. 5), were drawn. The Bland–Altmann method confirmed a high correlation between parameter values due to the slight deviation obtained in the mean values and the difference between them: All values were drawn in the limits of agreement (LoA ± 1.96 standard deviation).

## Discussion

Our study shows almost perfect inter-reader reliability for MD, FA, and RD on three different MR scanner and overall no statically significance differences among the three different vendors. A slightly decrease of inter-reader agreement was detected on Skyra MR for FA measurement in the right thigh. We suppose that this is due to the inclusion of tiny fatty areas of the subcutaneous tissue within the ROI [19].

Similar to previous studies on the nervous system, in our study the DTI values showed slightly statistical differences among different muscles as reported in Additional file (1) [13, 15, 18–20]. We assessed highest FA (0.348; IQR: 0.097) on gracilis muscle and lowest FA on vastus intermedius (second observer GE FA = 0.229; IQR: 0.038). Nevertheless, we believe that these differences, albeit slightly statically different, are not clinically significant because there is no overlapping between our FA values and those reported in patients with spinal muscular atrophy or muscular dystrophies ranging from 0.7 to 0.41 [9, 23–25]. Our results support the findings found by Fourè and colleagues who reported some differences among the FA values of the muscles of the lower limb [26]. However, values of the different muscles reported in this study are slightly different compared to ours. We believe that value discrepancies between the two studies may be due to different magnetic fields strength that determines higher SNR provided by acquisition on 7 T which improve fibre tracking compared to 3 T [27, 28].

Another study conducted on ten volunteers showed good reproducibility on both 3 and 7 T MR (Siemens Healthcare GmbH, Erlangen, Germany) with an SNR increase in the 7 T MR of up to 111% [12]. However, on the 7 T MR, the authors found higher FA and lower MD values in the soleus muscle, while the results of the remaining muscle compartments did not show significant statistical difference of the quantitative values between the MR. The authors justified the heterogeneity of the muscular values as the results of the same effect described by Polders et al., who found a higher uncertainty in peripheral areas of the brain on 7 T [29].

Interestingly, one study conducted on 18 healthy subjects on a single 3 T MR (Philips Medical Systems, Best, Netherlands) on the entire lower leg [30] and reported a statistically significant intra-muscle difference in FA between the origin of the muscle and the muscle belly. This is probably due to different chemical–physical properties of the actin-myosin components and the different amount and organization of collagen fibres.

M-DTI may be influenced by contraction and activity. Muscle contraction induces muscle fibre shortening and increases cross-sectional area (CSA), producing higher MD and lower FA values. Mazzoli et al. [31] performed MRI examinations of the lower leg on five volunteers during muscle contraction and found lower FA values of anterior tibialis in dorsiflexion compared with plantarflexion contraction and no contraction. The assessment of DTI values during muscle contraction is complex due to the length of current MRI sequences, which do not allow for constant, homogeneous fibre contraction. For this reason, we preferred to perform our MR examinations during muscle rest state. However, it is possible that in the future, with the increasingly widespread use of ultra-fast MRI sequences, the evaluation of muscle contraction will soon become available [32].

M-DTI may be influenced by activity, as well [33]. Hooijmans and colleagues described higher DTI values in upper legs muscles of 12 marathon runners in the post-marathon acquisition. The increase of MD and the decrease of FA are related to interstitial oedema and the alteration of diffusivity cellular barriers caused by muscle micro-trauma. These higher values returned to baseline (i.e. those values observed in the pre-marathon phase at the follow-up) after 3 weeks. MD and RD values are the first to return to the resting phase values, while FA values show a more prolonged alteration. This is the reason why we acquired MR examinations on the same days (within 6 h), without any sport activities performed the 4 weeks before the MR examination [34].

The first limitation of our study is the small sample size of volunteers, anyhow we assessed a large amount of data. Second, MR protocol parameters are not perfectly identical among the three MR scanners, because vendor-specific characteristics prevented us from applying exactly the same parameters for all the MR. However, other authors have used coils with different numbers of channels and DTI sequences with slightly different parameters to evaluate inter-observer and intra-observer agreement on the brain, obtaining promising results [13]. Moreover, the good results obtained with some parametric differences indirectly allow to obtain an even more significant inter-vendor agreement for clinical applications.

We believe that these reasons would make this sequence even more usable in clinical practice.

However, other studies with a larger sample of healthy volunteers are needed to confirm this claim.

## Conclusions

Our results highlight the inter-vendor and inter-reader reproducibility of m-DTI values, and we strongly believe that the use of this sequence should be more included in the MRI protocols during daily clinical practice for the evaluation of MSK pathologies.

## Supplementary Information

**Additional file 1**: **Table S1**. DTI values of each single muscle ROI of the arms of both observers. **Table S2**. DTI values of each single muscle ROI of the legs of both observers. **Table S3**. DTI values of each single muscle ROI of the thighs of both observers.

## Data Availability

The datasets used and/or analyzed during the current study are available from the corresponding author on reasonable request.

## Abbreviations

CSA: Cross-sectional area
FA: Fractional anisotropy
ICC: Intra-class correlation coefficient.
IQR: Interquartile ranges
MD: Mean diffusivity
M-DTI: Muscle diffusion tensor imaging
MR: Magnetic resonance
MSK: Musculoskeletal
RD: Radial diffusivity
ROIs: Muscle regions of interest
T: Tesla
