# Supplementary material for: Inter-vendor and inter-observer reliability of diffusion tensor imaging in the musculoskeletal system: a multiscanner MR study
Source: Insights Imaging. 2023 Feb 9;14:32. doi: 10.1186/s13244-023-01374-0 (PMC9911574; doi:10.1186/s13244-023-01374-0)
Supplement: Supplementary file 1 — Additional file 1: Table S1. DTI values of each single muscle ROI of the arms of both observers. Table S2. DTI values of each single muscle ROI of the legs of both observers. Table S3. DTI values of each single muscle ROI of the thighs of both observers. [file 13244_2023_1374_MOESM1_ESM.pdf]

ELECTRONIC SUPPLEMENTARY MATERIAL

Inter-Vendor and Inter-Observer Reliability of Diffusion Tensor Imaging in the musculoskeletal system: a Multiscanner MR Study

Insights Imaging (2023) Chianca V, Albano D, Rizzo S, Maas M, Sconfienza LM, Del Grande F

Table S1

	Observer 1							Observer 2						
	GE		Philips		Siemens		p-value	GE		Philips		Siemens		p-value
	Median	IQR	Median	IQR	Median	IQR		Median	IQR	Median	IQR	Median	IQR	
**Mean Diffusivity**														
Long head of triceps brachii	1.529	0.288	1.370	0.314	1.424	0.293	0.0001	1.524	0.282	1.375	0.262	1.425	0.242	0.0202
Median head of triceps brachii	1.482	0.263	1.452	0.165	1.460	0.099	0.3532	1.447	0.250	1.414	0.252	1.452	0.167	0.7933
Lateral head of triceps brachii	1.525	0.288	1.394	0.287	1.460	0.301	0.3840	1.526	0.315	1.415	0.297	1.464	0.319	0.2109
Biceps brachii	1.519	0.210	1.371	0.216	1.462	0.675	0.0381	1.50	0.213	1.373	0.199	1.527	0.432	0.3840
Coraco brachialis	1.496	0.222	1.423	0.194	1.421	0.286	0.3532	1.50	0.213	1.427	0.180	1.374	0.408	0.4917
**RA**														

Long head of triceps brachil	0.010	0.002	0.010	0.002	0.010	0.002	0.9364	0.010	0.002	0.009	0.002	0.010	0.002	0.9161
Median head of triceps brachil	0.010	0.001	0.010	0.002	0.009	0.001	0.1187	0.009	0.001	0.010	0.002	0.009	0.001	0.3197
Lateral head of triceps brachil	0.010	0.002	0.010	0.001	0.010	0.002	0.7393	0.010	0.001	0.010	0.001	0.009	0.001	0.3512
Biceps brachil	0.011	0.002	0.011	0.002	0.011	0.002	0.8435	0.011	0.002	0.011	0.001	0.011	0.003	0.9300
Coraco brachialis	0.012	0.004	0.012	0.002	0.012	0.003	0.4884	0.012	0.003	0.012	0.001	0.012	0.003	0.7502
**FA**														
Long head of triceps brachil	0.312	0.049	0.323	0.045	0.323	0.032	0.0775	0.313	0.038	0.298	0.060	0.323	0.035	0.3532
Median head of triceps brachil	0.299	0.030	0.309	0.037	0.302	0.022	0.7335	0.296	0.043	0.305	0.025	0.301	0.040	0.2737
Lateral head of	0.307	0.052	0.319	0.035	0.321	0.050	0.3840	0.296	0.035	0.322	0.040	0.322	0.036	0.0001

triceps brachil														
Biceps brachil	0.340	0.037	0.356	0.036	0.375	0.051	0.0007	0.342	0.039	0.356	0.034	0.369	0.034	< 0.0001
Coraco brachialis	0.353	0.070	0.386	0.055	0.377	0.055	0.0927	0.345	0.067	0.368	0.055	0.393	0.048	0.0039
**RD**														
Long head of triceps brachil	1.280	0.293	1.278	0.278	1.117	0.250	0.0039	1.132	0.220	1.228	0.299	1.218	0.243	0.0695
Median head of triceps brachil	1.236	0.243	1.203	0.198	1.232	0.132	0.3840	1.199	0.188	1.245	0.106	1.233	0.136	0.7933
Lateral head of triceps brachil	1.285	0.288	1.299	0.330	1.195	0.236	0.3840	1.166	0.203	1.234	0.262	1.262	0.294	0.2109
Biceps brachil	1.229	0.181	1.219	0.196	1.140	0.163	0.2109	1.155	0.149	1.219	0.795	1.269	0.724	0.2109
Coraco brachialis	1.218	0.179	1.236	0.159	1.138	0.167	0.1228	1.181	0.183	1.178	0.237	1.133	0.379	0.2839

Table S2

	Observer 1							Observer 2						
	GE		Philips		Siemens		p-value	GE		Philips		Siemens		p-value
	Median	IQR	Median	IQR	Median	IQR		Median	IQR	Median	IQR	Median	IQR	
**Mean Diffusivity**														
Medial gastrocnemius	1.560	0.170	1.536	0.241	1.533	0.248	0.2737	1.558	0.134	1.536	0.138	1.573	0.229	0.2839
Lateral gastrocnemius	1.546	0.125	1.535	0.280	1.687	0.249	0.3532	1.522	0.143	1.552	0.280	1.678	0.249	0.0310
Soleus	1.693	0.380	1.495	0.227	1.790	0.376	0.0202	1.786	0.416	1.455	0.209	1.768	0.337	0.0381
Anterior tibialis	1.633	0.344	1.608	0.133	1.662	0.322	0.0927	1.565	0.319	1.640	0.140	1.641	0.253	0.3532
Peroneal muscle	1.651	0.104	1.540	0.174	1.663	0.397	0.0381	1.653	0.109	1.551	0.175	1.670	0.340	0.0103
Posterior tibialis	1.489	0.242	1.553	0.380	1.577	0.147	0.2109	1.471	0.336	1.573	0.357	1.607	0.214	0.4917
Flexor digitorum longus	1.448	0.324	1.739	0.400	1.413	0.365	0.1228	1.475	0.323	1.671	0.454	1.374	0.292	0.2488
Flexor hallucis longus	1.580	0.378	1.753	0.347	1.785	0.716	0.3532	1.581	0.458	1.819	0.279	1.758	0.417	0.0695
**FA**														
Medial gastrocnemius	0.299	0.060	0.288	0.034	0.281	0.051	0.1444	0.302	0.044	0.288	0.044	0.282	0.054	0.3840

Lateral gastrocnemius	0.295	0.035	0.272	0.057	0.298	0.058	0.0192	0.306	0.030	0.274	0.051	0.296	0.043	0.0010
Soleus	0.283	0.049	0.279	0.064	0.265	0.020	0.1444	0.280	0.039	0.266	0.058	0.272	0.042	0.4917
Anterior tibialis	0.294	0.062	0.277	0.074	0.301	0.071	0.8716	0.277	0.069	0.274	0.064	0.293	0.047	0.0927
Peroneal muscle	0.351	0.081	0.307	0.079	0.321	0.060	0.0010	0.322	0.075	0.308	0.077	0.313	0.069	0.0039
Posterior tibialis	0.331	0.030	0.334	0.018	0.337	0.038	0.9803	0.336	0.034	0.328	0.021	0.325	0.025	0.6860
Flexor digitorum longus	0.324	0.072	0.331	0.093	0.317	0.076	0.1228	0.338	0.072	0.325	0.083	0.329	0.077	0.0310
Flexor hallucis longus	0.315	0.030	0.299	0.043	0.319	0.048	0.7737	0.314	0.026	0.301	0.033	0.307	0.037	0.2717
RD														
Medial gastrocnemius	1.328	0.154	1.308	0.184	1.314	0.236	0.5780	1.313	0.209	1.266	0.259	1.302	0.239	0.2737
Lateral gastrocnemius	1.291	0.123	1.281	0.102	1.335	0.264	0.2737	1.345	0.287	1.446	0.206	1.409	0.179	0.0422
Soleus	1.486	0.327	1.542	0.377	1.249	0.268	0.0072	1.246	0.182	1.574	0.325	1.542	0.253	0.0116
Anterior tibialis	1.406	0.285	1.358	0.295	1.373	0.136	0.1766	1.375	0.118	1.421	0.300	1.421	0.243	0.1766
Peroneal muscle	1.347	0.140	1.365	0.111	1.303	0.160	0.3840	1.300	0.155	1.366	0.295	1.414	0.255	0.0927
Posterior tibialis	1.219	0.248	1.218	0.365	1.324	0.308	0.3098	1.348	0.325	1.342	0.180	1.337	0.236	0.2737
Flexor digitorum longus	1.230	0.257	1.238	0.274	1.447	0.257	0.2109	1.419	0.348	1.149	0.384	1.123	0.318	0.2109

Flexor hallucis longus	1.338	0.376	1.346	0.440	1.480	0.245	0.3532	1.547	0.202	1.489	0.518	1.495	0.319	0.0695

Table S3

	Observer 1							Observer 2						
	GE		Philips		Siemens			GE		Philips		Siemens		
	Median	IQR	Median	IQR	Median	IQR	p-value	Median	IQR	Median	IQR	Median	IQR	p-value
**Mean Diffusivity**														
Rectus femoris	1.601	0.222	1.546	0.245	1.164	0.638	0.0519	1.599	0.234	1.519	0.270	1.142	0.758	0.0072
Medialis vastus	1.618	0.315	1.629	0.331	1.433	0.336	0.0202	1.655	0.311	1.670	0.282	1.401	0.301	0.0016
Lateralalis vastus	1.723	0.378	1.644	0.138	1.557	0.462	0.7943	1.712	0.403	1.667	0.197	1.574	0.435	0.5780
Intermedius vastus	1.672	0.255	1.642	0.143	1.733	0.258	0.4180	1.644	0.245	1.645	0.114	1.750	0.362	0.7737
Sartorius	1.452	0.352	1.447	0.307	1.427	0.301	0.7335	1.400	0.322	1.448	0.267	1.430	0.273	0.9262
Gracilis	1.324	0.257	1.501	0.273	1.454	0.207	0.3532	1.330	0.286	1.514	0.280	1.350	0.297	0.2109
Biceps femori	1.496	0.214	1.592	0.104	1.749	0.364	0.0422	1.505	0.211	1.603	0.101	1.760	0.370	0.0039

Semitendinosus	1.558	0.205	1.545	0.202	1.747	0.130	0.0018	1.581	0.217	1.564	0.204	1.801	0.167	0.0103
Semimembranosus	1.688	0.205	1.542	0.245	1.686	0.244	0.4917	1.642	0.198	1.520	0.280	1.726	0.179	0.0103
**FA**														
Rectus femoris	0.264	0.089	0.305	0.097	0.281	0.104	0.4180	0.258	0.086	0.304	0.087	0.282	0.108	0.3532
Medialis vastus	0.251	0.041	0.271	0.048	0.271	0.064	0.0361	0.253	0.043	0.274	0.053	0.271	0.072	0.4917
Lateralalis vastus	0.283	0.045	0.286	0.047	0.280	0.041	0.4662	0.289	0.060	0.283	0.037	0.283	0.054	0.5780
Intermedius vastus	0.235	0.025	0.233	0.048	0.232	0.040	0.6803	0.229	0.038	0.241	0.037	0.236	0.040	0.1817
Sartorius	0.320	0.105	0.347	0.097	0.331	0.120	0.7737	0.325	0.087	0.348	0.105	0.347	0.112	1.0000
Gracilis	0.325	0.059	0.348	0.068	0.315	0.064	0.7933	0.335	0.070	0.338	0.083	0.350	0.087	0.5780
Biceps femori	0.271	0.025	0.272	0.032	0.259	0.026	0.2737	0.266	0.039	0.271	0.052	0.258	0.033	0.3532
Semitendinosus	0.303	0.038	0.284	0.031	0.279	0.028	0.1557	0.300	0.038	0.290	0.035	0.282	0.039	0.1766
Semimembranosus	0.263	0.026	0.256	0.036	0.262	0.047	0.3532	0.267	0.037	0.260	0.041	0.251	0.038	0.2488

**RD**														
Rectus femoris	1.394	0.167	1.378	0.240	1.293	0.260	0.0575	1.282	0.277	1.017	0.665	0.997	0.760	0.0310
Medialis vastus	1.405	0.304	1.452	0.300	1.434	0.356	0.0202	1.461	0.312	1.245	0.285	1.216	0.253	0.0016
Lateralalis vastus	1.489	0.316	1.487	0.298	1.389	0.169	0.1766	1.416	0.227	1.328	0.379	1.326	0.379	0.3532
Intermedius vastus	1.459	0.219	1.444	0.214	1.446	0.128	0.8716	1.445	0.091	1.523	0.317	1.556	0.361	0.5369
Sartorius	1.195	0.221	1.183	0.247	1.206	0.282	0.7335	1.232	0.314	1.141	0.279	1.136	0.233	0.9262
Gracilis	1.138	0.256	1.062	0.239	1.213	0.219	0.7933	1.207	0.236	1.214	0.187	1.134	0.249	0.4917
Biceps femori	1.280	0.208	1.303	0.212	1.356	0.091	0.0276	1.392	0.107	1.487	0.274	1.504	0.312	0.0016
Semitendinosus	1.312	0.192	1.341	0.225	1.314	0.177	0.0012	1.324	0.184	1.465	0.150	1.495	0.102	0.0381
Semimembranosus	1.469	0.130	1.419	0.154	1.373	0.236	0.5780	1.335	0.267	1.485	0.178	1.523	0.135	0.0235
